# Why Do Intrauterine Exposure to Air Pollution and Cigarette Smoke Increase the Risk of Asthma?

**DOI:** 10.3389/fcell.2020.00038

**Published:** 2020-02-05

**Authors:** Baoming Wang, Hui Chen, Yik Lung Chan, Gang Wang, Brian G. Oliver

**Affiliations:** ^1^School of Life Sciences, Faculty of Science, University of Technology Sydney, Sydney, NSW, Australia; ^2^Woolcock Institute of Medical Research, The University of Sydney, Sydney, NSW, Australia; ^3^Department of Respiratory and Critical Care Medicine, Clinical Research Centre for Respiratory Disease, West China Hospital, Sichuan University, Chengdu, China

**Keywords:** asthma, fetus, placental, smoking, particulate matter

## Abstract

The prevalence of childhood asthma is increasing worldwide and increased *in utero* exposure to environmental toxicants may play a major role. As current asthma treatments are not curative, understanding the mechanisms underlying the etiology of asthma will allow better preventative strategies to be developed. This review focuses on the current understanding of how *in utero* exposure to environmental factors increases the risk of developing asthma in children. Epidemiological studies show that maternal smoking and particulate matter exposure during pregnancy are prominent risk factors for the development of childhood asthma. We discuss the changes in the developing fetus due to reduced oxygen and nutrient delivery affected by intrauterine environmental change. This leads to fetal underdevelopment and abnormal lung structure. Concurrently an altered immune response and aberrant epithelial and mesenchymal cellular function occur possibly due to epigenetic reprograming. The sequelae of these early life events are airway remodeling, airway hyperresponsiveness, and inflammation, the hallmark features of asthma. In summary, exposure to inhaled oxidants such as cigarette smoking or particulate matter increases the risk of childhood asthma and involves multiple mechanisms including impaired fetal lung development (structural changes), endocrine disorders, abnormal immune responses, and epigenetic modifications. These make it challenging to reduce the risk of asthma, but knowledge of the mechanisms can still help to develop personalized medicines.

## Introduction

Asthma is a disease that generally affects 5–20% of children globally (Global Asthma Network, 2018; Enilari and Sinha, 2019). It is a complex condition in which symptoms are mainly caused by bronchoconstriction (Thomson and Hasegawa, 2017). Airway constriction occurs rapidly in response to a variety of inhaled substances, for example, allergens such as pollen and house dust mite, and environmental sources such as dust and smoke, which usually can be fully or partially reversed by bronchodilators. Pathologically it is defined by airway remodeling, typified by increased smooth muscle and epithelial layer thickness, and increased numbers of inflammatory cells. However, the type of inflammation varies. For example, sputum based phenotyping of inflammation categorizes people into eosinophilic, neutrophilic, or paucigranulocytic asthma. The other factors that can add to the complexity of asthma including the age of onset, etiological cause (if known), co-existence of other respiratory diseases, comorbidities, the degree of reversibility, and the ability for the symptoms being effectively controlled by pharmaceutical interventions.

The susceptibility to asthma is complex, which involves both genetic susceptibility, environmental insults (both pre and post birth), and is further complicated by asthma symptoms initiating and sometimes ceasing at different ages, as well as differences in asthma prevalence between the male and female sexes (Carey et al., 2007b).

It is known that boys are more susceptible than girls before puberty, but less than girls after puberty. Many theories exists to explain this phenomena including: dysnapsis due to different sized lungs in boys and girls, increased allergy (more IgE production in boys), different innate and adaptive immune responses in boys and girls, and the influence of sex hormones (Shames et al., 1998; Papadopoulos et al., 2004; Mohammad et al., 2016). The incidence of asthma is also related to the use of life saving medical interventions in premature and newborn children such as oxygen supplementation or mechanical ventilation due to physical permanent damage to the newborn’s lungs (Davidson and Berkelhamer, 2017).

However, it has increasingly been recognized that certain factors during the intrauterine period affects childhood asthma susceptibility. In particular, maternal smoking (MSE) and particulate matter (PM) exposure (Burnett et al., 2014; Thacher et al., 2014), are the best described/researched *in utero* challenges which affect asthma susceptibility. This review will discuss the current understanding of multiple mechanisms underlying these two factors, which may help to develop personalized medicines.

## Epidemiology of Asthma

The prevalence of allergic disorders has been rising since the early 1980s. The average global rate of allergic disorders is 22%, ranging from 15 to 35% of the population in different countries (Warner et al., 2006). According to the WHO, the number of children with asthma is around 14% globally (Asthma fact sheet World Health Organisation, 2017). Severe asthma is common in children. A recent study reported that the prevalence of severe asthma was 4.9% in 6–7 years old children, however, the incidence was increased to 6.9% in 13–14 years olds. These phenomena demonstrated that age is an important factor for the onset of asthma (Lai et al., 2009).

Environmental toxicant exposure during pregnancy is a significant factor that has been shown to increase the incidence of asthma (Crinnion, 2012). In particular, MSE is the largest modifiable risk factor for the development of asthma. Although the harmful effect of smoking is well-known in the general public, smoking mothers find it difficult to quit due to nicotine addiction, even during pregnancy when nicotine metabolism is faster than non-pregnant status (Taghavi et al., 2018). A systematic review and meta-analysis in the Lancet showed that the top 3 countries with the highest smoking rate during pregnancy are Ireland (38.4%), Uruguay (29.7%), and Bulgaria (29.4%) (Lange et al., 2018). Even in Australia where anti-smoking legislation is one of the most aggressive in the world, the smoking rate in pregnant women is 11.7% (Laws et al., 2010).

Epidemiological studies have demonstrated a dose-dependent increase in asthma risk in offspring due to MSE (Table 1). Currently, several cohort studies have confirmed the association between MSE and asthma risk in the offspring (Strachan and Cook, 1998; Gilliland et al., 2001; Doherty et al., 2009; Burke et al., 2012). For example, a birth cohort study has found that women smoking during pregnancy could increase asthma incidence in the offspring with an adjusted hazard ratio of 1.79 (95% CI 1.20–2.67) (Grabenhenrich et al., 2014). The same outcome has been found in another cohort study where MSE during pregnancy caused higher asthma risk in the child in the first year of life with an odds ratio (OR) of 1.83 (Gold et al., 1999). Similarly, a systematic review of 14 studies revealed a wheezing [OR 1.41 (95% CI 1.19–1.67)] and asthma risk [OR 1.85 (95% CI 1.35–2.53)] in 2 years old and younger children, followed by a higher asthmatic risk in 5–18 years old children [OR 1.23 (95% CI 1.12–1.36)] caused by smoking during pregnancy (Burke et al., 2012). One study found a strong asthma risk in 14 year old girls whose mothers smoked during pregnancy, however this was not found in boys (Alati et al., 2006); whereas a different study found that boys at the age of 11 are more susceptible to the maternal and postnatal secondhand smoke (Hu et al., 2017). These differences might be related to the changes in asthma prevelance in boys and girls around puberty.

**TABLE 1:** Maternal smoking during pregnancy and the risk of asthma in children.

**Smoking exposure**	**Age**	**Relative risk Odds ratio (95% CI)**		**References**
		**Male**	**Female**	
Smoker at some stage	14 years	1.15 (1.01–1.72)	1.25 (0.85–1.22)	Alati et al., 2006
>20 cigarettes (early and late)	14 years	0.57 (0.20–1.60)	1.09 (0.47–2.51)	Alati et al., 2006
Total of 1–9 cigarettes/day	4–16 years	1:19 (0.98–1.43)		Thacher et al., 2018
<10 Cigarettes per day	7 years	1.20 (1.04–1.38)		Jaakkola and Gissler, 2004
Total of ≥10 cigarettes/day	<5 years	1.68 (1.10–2.58)		Martinez et al., 1992
>10 Cigarettes per day	7 years	1.31 (1.09–1.58)		Jaakkola and Gissler, 2004
Total of ≥10 cigarettes/day	4–16 years	1:66 (1.29–2.15)		Thacher et al., 2018
Smoking during pregnancy	First 3 years	1.88 (1.14–3.12)		Murray et al., 2004
Smoking during pregnancy	4–6 years	1.65 (1.18–2.31)		Neuman et al., 2012
Smoking during pregnancy	2–7 years	1.7 (1.2–2.2)		Harju et al., 2016
Smoking during pregnancy	5–9 years	0.97 (0.51–1.84)		Sherman et al., 1990
Smoking during pregnancy	14 years	1.49 (0.91–2.45)		Hollams et al., 2014
Smoking during pregnancy	7–16 years	0.99 (0.78–1.25)		Strachan et al., 1996

Around 91% of the world’s population are living in the areas where the levels of air pollutants exceed the WHO limits (Balakrishnan et al., 2019). Epidemiological studies demonstrated a strong association between pulmonary disease and particular matter (PM) exposure (Burnett et al., 2014). Compared to cigarette smoking which can be avoided through quitting, the dangers of airborne pollution are hard to avoid in heavily polluted countries, such as China and India. In China, 74,000 premature deaths were attributed to PM_2__.__5_ exposure in the year 2013 (Ji et al., 2019). It was estimated that 22% of these deaths could have been avoided if indoor PM_2__.__5_ level met National Class I standards (Ji et al., 2019).

There are many different types of airborne pollution, but simplistically these can be divided into gasses and particulate matter (PM). PM is considered as particularly dangerous as respirable particles can remain airborne over large distances.

As shown in Table 2, prenatal PM exposure is also associated with childhood asthma. A cohort study found that prenatal PM_10_ exposure could cause pulmonary function changes with higher minute ventilation in newborns (Latzin et al., 2009). Another birth cohort study including pre-school and school-age children demonstrated that prenatal PM_10_ exposure increased the risk of developing asthma in both age groups, especially for those pregnant mothers who lived near the highways (Sbihi et al., 2016). The correlation between maternal PM exposure and asthma risk in different genders was also investigated. High levels of PM_2__.__5_ exposure during mid-gestation increased the development of asthma by the age of 6 years in boys, but not in girls (Hsu et al., 2015). The above evidence indicates that maternal PM exposure during pregnancy has similar effects to MSE in terms of increasing the risks of developing asthma in childhood.

**TABLE 2:** Maternal PM exposure and the development of asthma in offspring.

**Pollutant**	**Age**	**Concentration increase**	**Relative risk**	**References**
PM_2__.__5_	6 years	1.7 μg/m^3^ (per IQR)	1.15 (1.03–1.26)	Lee A. et al., 2018
PM_2__.__5_	3–4 years	1 μg/m^3^ (exposure interval)	0.95 (0.91–1.00)	Clark et al., 2009
PM_2__.__5_	0–5 years	1.45 μg/m^3^ (per IQR)	0.99 (0.97–1.01)	Sbihi et al., 2016
PM_2__.__5_	6–10 years	1.46 μg/m^3^ (per IQR)	1.01 (0.97–1.06)	Sbihi et al., 2016
PM_2__.__5_	0–6 years	3.7 μg/m^3^ (per IQR)	1.01 (0.99–1.04)	Lavigne et al., 2018
PM_10_	3–6 years	12 μg/m^3^ (per IQR)	0.89 (0.68–1.16)	Deng et al., 2016
PM_10_	3–4 years	1 μg/m^3^ (exposure interval)	1.09 (1.05–1.13)	Clark et al., 2009
PM_10_	0–5 years	1.3 μg/m^3^ (per IQR)	1.12 (1.05–1.19)	Sbihi et al., 2016
PM_10_	6–10 years	1.36 μg/m^3^ (per IQR)	1.09 (0.96–1.24)	Sbihi et al., 2016

*IQR, interquartile range.*

The difference of asthma prevalence between boys and girls and the change in prevalence which occurs around puberty naturally gives credence to the involvement of sex hormones. Animal models of estrogen receptor knockouts suggests that estrogen promotes the development of the asthma (Carey et al., 2007a); while male mice lacking testosterone showed more severe asthma symptom (Yu et al., 2002). These studies help to explain why boys are more susceptible to asthma before puberty, and girls more susceptible after puberty. However, the etiology of asthma is complex and is multifactorial.

## The Role of Oxidative Stress in the Development of Asthma in Children

Various chemicals can be found in both cigarette smoke and PM. It is unlikely that a single chemical is responsible for all the adverse effects of *in utero* exposure to cigarette smoke or PM on lung health in the offspring. Cigarette smoke and PM are two major environmental sources of inhaled free radicals and strong oxidants. The balance between excessive oxidant activity and the antioxidant capacity can tip in favor of excess oxidants causing oxidative stress. However, it is important to note that the production of oxidants is necessary to maintain healthy cell function, and important in regulating processes such as inflammatory responses. Oxidative stress induces adverse effects in tissues. The developing fetus is highly vulnerable to oxidative stress injury, as the immune system remains immature during the prenatal period (Lee A. G. et al., 2018). Free radicals and chemicals inhaled during MSE and maternal PM exposure can pass the blood-placental barrier to directly increase the level of oxidative stress in the offspring. Therefore, we propose the first common and prominent mechanism underlying these two factors to induce pathological changes in the offspring is oxidative stress.

Our previous studies in mice have repeatedly shown that MSE can reduce the level of endogenous antioxidant Manganese Superoxide Dismutase in the brain, kidney, and lungs of adult offspring accompanied by increased Reactive Oxygen Species (ROS) levels in those organs; interestingly, antioxidant supplementation during pregnancy could completely or partially reverse the adverse effects on those organs induced by MSE (Chan et al., 2017; Sukjamnong et al., 2017, 2018). The endogenous antioxidant enzyme system is established in the second and third trimester of pregnancy and continues to develop in early childhood (Fanucchi, 2004). Interestingly, lung development also matures in the early postnatal period, suggesting that the antioxidant system may protect early life lung development from the adverse impacts of environmental oxidant pollutants (Pinkerton and Harding, 2014). After all, the function of the respiratory system is vital for survival immediately after birth. Vitamin C is an antioxidant which contributes to cellular antioxidant defense (Preston, 1991; Tous et al., 2019). A study in pigs found that vitamin C deficiency during pregnancy could cause brain damage in the offspring (Schjoldager et al., 2015). Giving smoking women vitamin C during pregnancy was shown to improve lung function (better airflow and less wheezing) in children during the first year of life (McEvoy et al., 2014). This again provided evidence that oxidative stress and insufficient capacity of antioxidants play a key role in organ dysfunction in the offspring due to MSE. PM consists of metals and endotoxins (polycyclic aromatic hydrocarbons) which also can generate ROS (Billah, 2015) and produce oxidative damage (Valavanidis et al., 2006). Therefore, the pathways associated with oxidative stress are regarded as playing an important role in inducing adverse respiratory outcomes after the exposure to environmental pollutants (Breland et al., 2002; Romieu et al., 2008).

*In utero*, any adverse effects that occur during fetal development can have long-lasting negative influences on organ development and later function after birth (Aycicek et al., 2005; Noakes et al., 2007). In fact, local tissue oxidative stress and injury due to the imbalance between free radicals and antioxidant capacity is a key factor in asthma pathogenesis. As such we propose that oxidative stress is the pathological insult that drives changes in the intrauterine environment and disturbs normal fetal development which subsequently increases the risks of developing asthma. It is also worth noting that maternal smoking is a strong risk factor for miscarriage, a process also linked to oxidative stress (Stone et al., 2014).

## Intrauterine Growth Restriction – the Barker Hypothesis

In 1990, the epidemiologist David Barker presented his hypothesis which linked chronic and degenerative diseases, such as heart disease, to the poor intrauterine environment caused intrauterine growth retardation (IUGR), low birth weight, and premature birth. This theory inspired scientists and has been expanded to the other organ systems including the respiratory system (Zacharasiewicz, 2016). Numerous studies have confirmed that environmental toxicant exposure during pregnancy, such as cigarette smoke, can cause IUGR and subsequently abnormal lung development in the offspring (Zacharasiewicz, 2016). Nicotine is the most widely studied component in cigarette smoke due to its addictive effects. Early studies showed that cotinine, the stable metabolite of nicotine, can be found in fetal circulation and body fluids (Sabra et al., 2017). This indicates that chemicals in cigarette smoke can cross the blood-placental barrier and reach the fetus. A more recent study by Geelhoed et al. (2011) showed that MSE can decrease blood flow in the ascending aorta because of higher arterial resistance in the uterus, which can reduce the oxygen and nutrient delivery to the growing fetus resulting in IUGR. Inadequate nutrient availability in the developing fetus, especially during the periods of rapid lung growth, has been shown to induce lung developmental defects (Chen et al., 2004; McMullen et al., 2005) and respiratory morbidity in the offspring (Harding et al., 2004; Maritz et al., 2005). Animal studies have demonstrated a decrease in both alveolarization and vessel density in the lung of sheep with IUGR (Rozance et al., 2011).

## How Do MSE and Maternal PM Exposure Impact on Fetal Lung Development?

In brief, MSE can induce such effects in two ways: the direct influence on the developing fetus, and indirect effects on the fetoplacental unit. Recently, studies have demonstrated that a small fraction of the circulating nicotine in the mothers can cross the trophoblastic membrane and reach the unborn child, and as such cotinine can accumulate in the fetal circulation and fluids in measurable concentrations (Jauniaux and Burton, 1992; Jauniaux et al., 1999). Furthermore, a similar concentration of cotinine in both fetal lung tissue and blood was found, suggesting cotinine may bind to the receptors in the lung to directly affect fetal lung development (McEvoy and Spindel, 2017). Maternal air pollution exposure can also cause fetal growth restriction (Bonzini et al., 2010). Polycyclic aromatic hydrocarbons on the surface of PM can easily cross the blood-placental barrier and circulate in the fetal blood because of its small size (Jauniaux et al., 1999). Therefore, lung development in the fetus can be directly affected by the PM inhaled by the mothers.

The fetoplacental unit has a significant influence on fetal development. The damage to fetoplacental unit caused by maternal smoking can be seen during early pregnancy. For example, MSE significantly increases villous membrane thicknesses and trophoblastic layer in the placenta during the first trimester (Jauniaux and Burton, 1992). There are also signs of reduced capillary volume in placental vasculature in pregnant smokers (Burton et al., 1989). The consequence of reduced capillary volume is nutrient delivery decrement. Intrauterine nutrient deficiency has been suggested as the major factor contributing to fetal growth restriction and low birth weight due to MSE (Figueras et al., 2008). Low birth weight can increase the asthma risk in later life, evidenced by a meta-analysis including 1.1 million people (Xu et al., 2014). In rat models, maternal PM exposure was found to change placental morphology, and decrease placental weight, size and surface area (de Fátima Soto et al., 2017). Similar findings have also been confirmed in humans, where PM_10_ exposure can decrease placental weight with higher anti-angiogenic factors in cord blood (van den Hooven et al., 2012). As a result, increased vascular resistance can be predicted, which will reduce uteroplacental perfusion and lead to various maternal and fetal complications, such as low birth weight and miscarriage (Kaufmann et al., 2003; Ness and Sibai, 2006; Schlembach et al., 2007).

The abovementioned evidence indicates that MSE and maternal PM exposure during pregnancy can impair fetal lung development through a direct effect on the fetus and indirect influence on placental morphology and function. However, the molecular mechanisms underlying the increased risk of asthma due to MSE and maternal PM exposure are not well understood. In monkeys, MSE upregulated nicotinic acetylcholine receptors in the fetal lung, associated with lung function decline after birth (Sekhon et al., 1999, 2001). Several *in vitro* and *in vivo* animal models have also shown that both MSE and PM exposure during pregnancy affects the development of the neonatal immune system, lung structure, and lung function in the offspring, making them more susceptible to the development of asthma (Collins et al., 1985; Mauad et al., 2008). These will be discussed in greater detail later.

## The Development of Asthma in Children

### The Role of Altered Lung Structure

Just as discussed above, MSE and maternal PM exposure during pregnancy can result in oxidative stress, and cause nutrition deficiency resulting in IUGR, which eventually alters lung development and structure. Fetal lung development starts from embryo Weeks 3–5 when the laryngotracheal groove forms on the floor of the foregut and matures during the early postnatal year. Therefore, inhaled environmental toxicants by pregnant mothers may change lung morphology and function as early as gestational Weeks 5–17 when epithelial and smooth muscle cell differentiation takes place. Epidemiological evidence well supports this theory, where significant lung function impairment was found in the newborns of mothers who smoked during pregnancy or inhaled high levels of PM (Carlsen et al., 1997; Latzin et al., 2009). Such lung function disorders can last until later childhood (Jedrychowski et al., 1997, 2010). It needs to be noted that lung function deficiency in early life has been correlated with increased asthma incidence later on (Borrego et al., 2009).

Lung dysfunction after birth can be attributed to lung structural changes during fetal development. Animal studies have shown that both MSE and maternal PM exposure could decrease lung volume, alveoli number and mean linear intercept in the offspring as well as reduced alveolar–bronchiolar attachment points (Collins et al., 1985; Elliot et al., 2001; Mauad et al., 2008). Nicotine as the “addictive substance” in tobacco smoke has often been used in animal models to investigate the potential mechanisms underlying the adverse effects of maternal tobacco smoking. For example, increased airway collagen deposition and altered vascular structure were found in a monkey model after prenatal nicotine exposure (Sekhon et al., 1999, 2004). However, it is uncertain if these results can be translated to humans as nicotine replacement therapy during pregnancy has not been found to be associated with the same adverse outcomes as maternal cigarette smoking (Dhalwani et al., 2015) or nicotine administration in animal models (Sekhon et al., 1999, 2004). This suggests that the whole constituent of tobacco smoke is needed to study the mechanism in animals.

### The Role of Endocrine Disorders

Endocrine disruption during pregnancy is a potential cause of adverse pregnancy outcomes. Endocrine glands form an important part of the fetoplacental unit that can secrete a significant amount of hormones including the estrogen to support pregnancy. Estrogen plays a key role in regulating neuroendocrine homeostasis in the developing fetus and promotes Th2 immune cell development in the fetus (Xu et al., 2003; Wood, 2014). A human study demonstrated that abnormal estrogen level in pregnant mothers affects fetal development (Migliaccio et al., 1996). A reduction in estrogen and estrone (a weak estrogen) levels in the cord blood has been found if the mother smoked during pregnancy (Varvarigou et al., 2009). This is because smoking can produce an anti-estrogenic effect and induce androgenisation in pregnant mothers to disturb hormonal homeostasis (Håkonsen et al., 2014). Such changes may influence the risk of asthma in offspring (Rangaraj and Doull, 2003).

The evidence to prove the relationship between maternal PM exposure and its impact on endocrine homeostasis are scarce. It has been shown that the endocrine-disrupting chemicals (EDCs) on the surface of PM can disrupt sex hormone synthesis (Lauretta et al., 2019). Polycyclic aromatic hydrocarbons in both tobacco smoke and PM, can also affect steroidogenesis through inhibiting steroidogenic enzymes (Rocha Monteiro et al., 2000). However, there is no direct evidence suggesting the correlation between hormone change induced by maternal PM exposure and fetal lung development, neither is known about the risk of asthma in the offspring (Street et al., 2018).

However, the information collected from cord blood at birth can’t accurately reflect the changes in fetal lung development during particular sensitive windows of embryo development induced by MSE and Maternal PM exposure. Amniocentesis is an alternative method to measure hormone levels at different time points and explore endocrine disruption, but access is limited. Animal modeling may shed a light on the correlation between placental hormone changes and fetal lung development, as well as postnatal lung function and susceptibility to asthma. Future research can focus on this aspect to better understand the niche factors contributing to lung development and the risk of asthma.

### The Role of Epigenetic Programing

Programing is a term used to describe an altered phenotype due to changes in the *in utero* environment. Epigenetic programing describes stable inheritable phenotypic changes without the alteration in the DNA sequence. Such a process controls mRNA expression and protein production through changing the transcriptome, including DNA methylation and histone modifications. Mounting evidence has closely linked asthma to epigenetic programing due to intrauterine environmental changes. For example, asthma is also an inheritable disease (Eder et al., 2006). The parent-of-origin effect which is usually due to epigenetic mechanism, also shows a prominent influence on the development of asthma, e.g., asthmatic mothers are more likely to have offspring with asthma than the asthmatic fathers (Moffatt and Cookson, 1998). As mitochondrial DNA is 100% inherited from the mothers, epigenetic modification of this genome may largely contribute to this phenomenon. In addition, the fetal period is a vulnerable stage and thus very sensitive to environmental toxicant exposure, when maternal protection is vital. During embryogenesis, cells divide rapidly and therefore the genome is in a relatively unstable status. During this period, oxidative stress induced by environmental toxicant exposure may easily interrupt genomic duplication process (Foley et al., 2009), leading to abnormal epigenetic modifications or even mutation, rendering the fetus susceptible to future chronic diseases after birth, such as asthma.

In a cohort study on MSE, CpGs methylation has been found on genes responding to the pollutants in tobacco smoke in the newborns of smokers who smoked during pregnancy (Joubert et al., 2014). In addition, CpG methylation was also found in the genes involved in fetal development in cord blood by MSE, suggesting a mechanism by which MSE results in intrauterine underdevelopment (Joubert et al., 2014). Previous studies have shown that maternal PM exposure could alter DNA methylation in the offspring. Prenatal PM_10_ exposure induced superoxide dismutase 2 (SOD2) protomer methylation in cord blood cells (Zhou et al., 2019), which is related to phthalate and diisocyanate-induced asthma (Yucesoy et al., 2012; Wang and Karmaus, 2017). As the epigenetic changes are inheritable, they will change gene expression to affect normal embryo development and persist throughout life, resulting in the susceptibility to chronic diseases in later life (Montgomery and Ekbom, 2002). It may also result in the transfer of certain respiratory diseases to subsequent generations, such as asthma, establishing a family history. For a detailed review on epigenetic changes due to *in utero* oxidative challenges, please see Zakarya et al. (2019).

### The Role of the Immune Response

The mother’s immune system plays a central role in the protection of fetal development. The fetus and newborns need maternal antibodies (Ig) to protect them from infectious diseases (Niewiesk, 2014). Previous studies have shown that parental smoking and PM exposure increased Ig E levels in the cord blood (Valavanidis et al., 2006; Liu et al., 2007). MSE and maternal PM exposure can also alter immune responses through activating inflammatory macrophages and memory B cells in the offspring (Prins et al., 2012; Yoshida et al., 2012). These changes in immune responses suggest that MSE and maternal PM exposure can alter the innate and adaptive immune response in the offspring. In addition, MSE and maternal PM exposure have also been shown to delay the maturation of immune system (Ege et al., 2006; Noakes et al., 2006), which may also make such offspring more susceptible to allergic disorders.

Toll-like receptors (TLRs) play an important role in the neonatal immune response (Yoon, 2010). MSE can inhibit neonatal immune system maturation through impairing TLR mediated responses (such as TLR2 and TLR9) (Noakes et al., 2006). We also have similar observations in the brains of mice who are offspring which had MSE. At postnatal day 1, mRNA expression of TLR4 was decreased in the offspring from MSE compared to those from Sham-exposed mothers, suggesting suppressed immune response or delayed maturation of immune response (Chan et al., 2016). However, TLR4 mRNA expression was increased in 13 weeks old offspring which had MSE along with increased inflammatory cytokines expression (Chan et al., 2016), suggesting that MSE has a sustainable influence on the immune system leading to heightened inflammatory cytokines production. Maternal PM exposure could induce similar adverse effects. High levels of TLR2 and TLR4 expression were found in the human offspring and animals from mothers exposed to increased levels of PM during pregnancy (Ege et al., 2006).

Asthma is typified by T cell dysregulation, including Th1, Th2, and Th17 cells (Kaiko et al., 2008). In most asthmatic patients, accumulating evidence shows the suppression of Th1 cytokines (for example IFNγ) with higher Th2 cytokine expression (IL-4, IL-5, and IL-13) (Mazzarella et al., 2000). Furthermore, clinical data showed that allergic responses are more prevalent among the children who have developed attenuated Th1 responses during infancy (Shirakawa et al., 1997). Similar changes were found in animal studies. In pregnant C57BL/6 mice, intranasal exposure to diesel exhaust particles has been shown to increase the Th2 cell percentage in the bronchoalveolar lavage fluid with higher levels of pro-inflammatory cytokines (IL-4 and IL-5) in the offspring with asthma (Manners et al., 2014). MSE was also shown to increase Th2 cytokines (IL-4 and IL-5) and other pro-inflammatory cytokines (such as IL6) with suppressed Th1 cytokines (IFN-γ) due to reduced NK cell activities (Singh et al., 2011; Prins et al., 2012).

However, the immune response is complicated, and difficult to investigate from a broader spectrum. A study has found that PM_2__.__5_ exposure differentially impacts the immune system at different stages of gestation. High level of CD3 + and CD4 + lymphocytes and low percentage of CD19 + lymphocytes and NK cells can be found in the cord blood during the early gestation; however, the opposite changes with low level of CD3 + and CD4 + lymphocytes and high percentage of CD19 + lymphocytes and NK cells were found if PM exposure occurs during late gestation (Herr et al., 2010). These studies suggest that immune response has been programed by *in utero* exposure to air pollution, however, future studies are needed to fully understand the extent of the changes in this system.

## Conclusion and Perspectives

**FIGURE 1:**
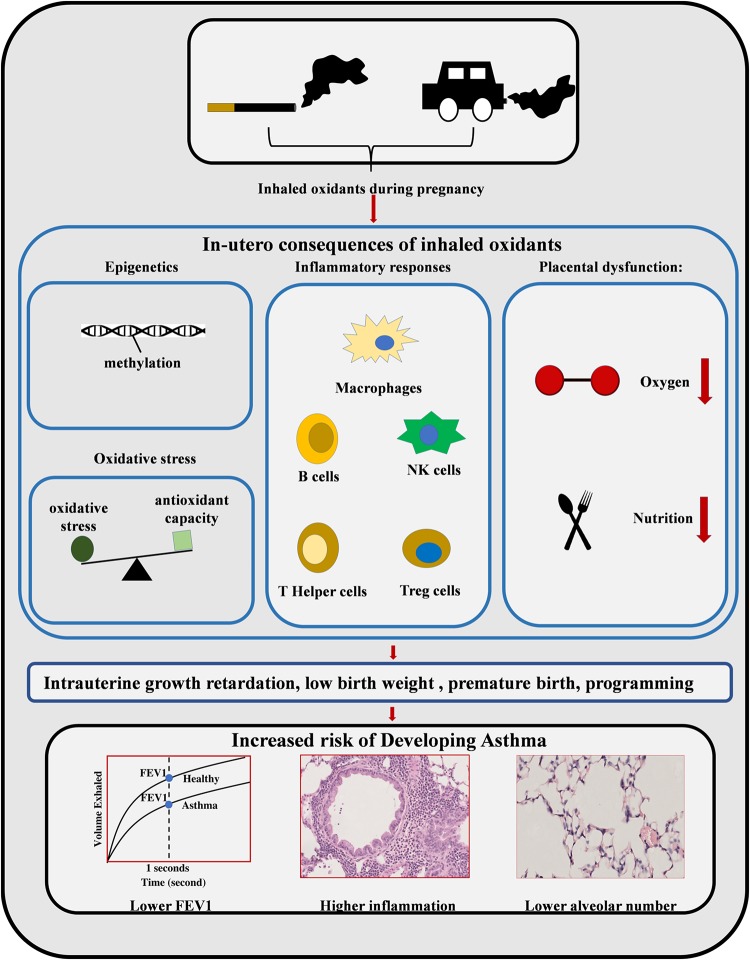
Maternal smoke exposure and maternal PM exposure can increase the rate of childhood asthma. MSE and maternal PM exposure can induce various adverse impacts on the fetus during different intrauterine developmental stages, such as DNA methylation, oxidative stress, inflammatory responses, and placental dysfunction. The resulting intrauterine growth retardation, low birth weight, and premature birth can increase the risk of childhood asthma with a lower alveolar number and reduced lung function, as well as increased lung inflammation.

**FIGURE 2:**
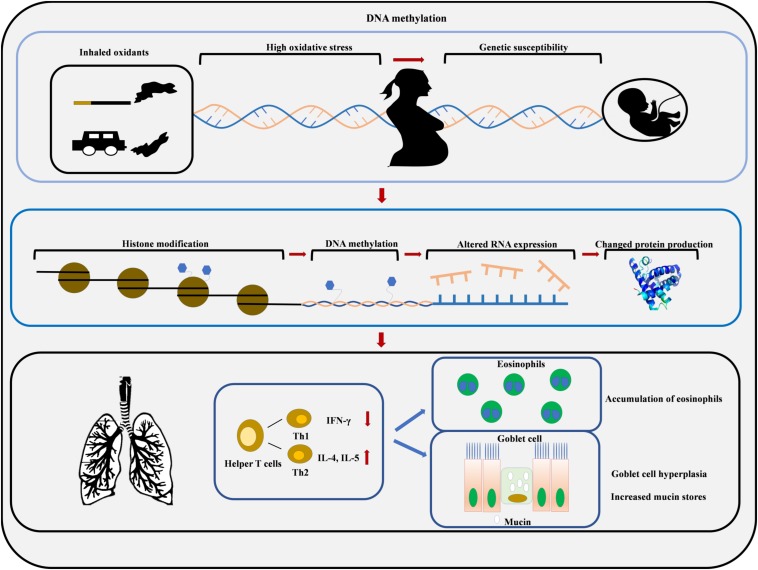
Maternal smoke exposure and maternal PM exposure increase oxidative stress in the womb which increases the risk of developing asthma due to the epigenetic modification of fetal DNA. Environmental toxicants can induce histone modifications and DNA methylation, which results in Th2 cytokine overproduction, eosinophils accumulation, goblet cell hyperplasia, and mucin hypersecretion.

**FIGURE 3:**
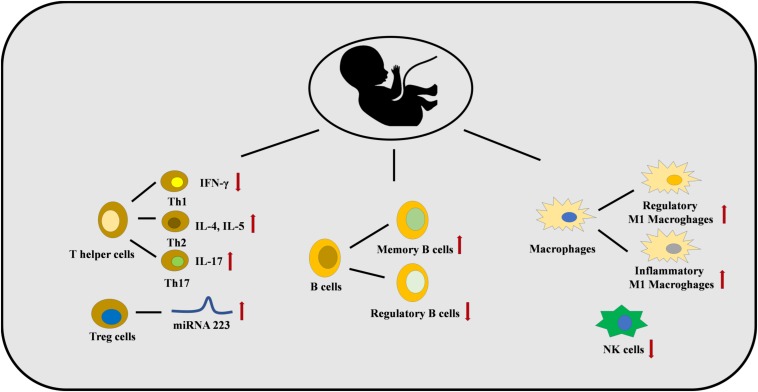
Maternal smoke exposure and maternal PM exposure can dysregulate the immune system in the fetus. The numbers of Th2 and Th17 cells are increased with a lower number of Th1 cells. This is caused by several epigenetic mechanisms, for example, miRNA 223 is increased in Treg cells. B cell and macrophages differentiation are also affected, and a lower number of NK cells are found.

In conclusion, cigarette smoking and PM exposure during pregnancy is detrimental to fetal development and increase the risk of childhood asthma (Table 3). As summarized in Figures 1–3, oxidants inhaled by the mother result in increased oxidative stress in the intrauterine environment. This results in persistent changes to both the structure of the lung and the epigenome, altering immune and endocrine systems. Collectively these changes increase the risk of childhood asthma. Although smoking cessation is preferred, the success rate remains low during pregnancy. Given the similarity between MSE and maternal PM exposure, antioxidant supplementation during pregnancy may be a plausible prophylactic strategy, which is yet to be confirmed by large clinical trials.

**TABLE 3:** Clinical evidence of the adverse impacts of MSE and maternal PM exposure.

**Pollutant**	**Sample collecting time (gestation)**	**Adverse impact**	**References**	
Maternal smoking	9–14 weeks	High villous membrane and trophoblastic layer thicknesses	Jauniaux and Burton, 1992	Placenta
Maternal smoking	–	Smaller villous capillaries and high basement membrane thickness	Van der Velde et al., 1983	
Maternal smoking	–	High villous membrane thickness	Burton et al., 1989	
Maternal smoking	28 ± 1 weeks	Decreased uterine artery volume	Castro et al., 1993	
Maternal smoking	1st trimester	More NK cells and macrophages, less regulatory T cells	Prins et al., 2012	Immune cells regulation
Maternal smoking	34th week	Lower Treg cell numbers	Herberth et al., 2014	
Maternal smoking	After delivery	Attenuated innate immune responses	Noakes et al., 2006	
Maternal smoking	During gestation	DNA methylation in cord blood cells	Joubert et al., 2016	Epigenetics
Maternal smoking	6–28 weeks infants	Lower antioxidant level and high oxidative stress level	Aycicek et al., 2005	Oxidative stress
Maternal smoking	3 months infants	Higher markers of oxidative stress	Noakes et al., 2007	
PM_10_	1st and 2nd-trimester	Lower Pro- and anti-angiogenic factors and PlGF	van den Hooven et al., 2012	Placenta
PM_2__.__5_	Early/late gestation	Higher CD3 + and CD4 + lymphocytes and lower CD19 + and NK cell number during early gestation, which were opposite in the late gestation	Herr et al., 2010	Immune cells regulation
PM_2__.__5_	After delivery	Higher GSTP1 methylation	Lee A. G. et al., 2018	Epigenetics
PM_2__.__5_	During gestation	Higher 3-NTp levels (oxidative stress)	Saenen et al., 2016	Oxidative stress

*GSTP1, Glutathione S-Transferase Pi 1; 3-NTp, 3-nitrotyrosine; MSE, maternal smoke exposure; NK cells, natural killing cells; PlGF, placental growth factor; PM, particulate matter; Treg cells, T regular cells.*

## Author Contributions

BW, HC, and BO designed and wrote the manuscript. YC and GW contributed to the grammar checking.

## Conflict of Interest

The authors declare that the research was conducted in the absence of any commercial or financial relationships that could be construed as a potential conflict of interest.
